# Supplementary material for: Krill Protein Hydrolysate Provides High Absorption Rate for All Essential Amino Acids—A Randomized Control Cross-Over Trial
Source: Nutrients. 2021 Sep 14;13(9):3187. doi: 10.3390/nu13093187 (PMC8465607; doi:10.3390/nu13093187)
Supplement: Supplementary file 1 [file nutrients-13-03187-s001.zip › nutrients-1305556-supplementary.pdf]

Supplementary Table S1. Amino acid profile of test proteins per 100g

	Krill Protein Hydrolysate	Whey protein isolate	Soy protein isolate
Macronutrient composition			
Energy (kcal)	375	367	392
Protein¤ (g)	93.0	87	88
Fat (g)	<0.4	0.9	3.8
Carbohydrates (g)	0.0	3.0	0.0
Calcium content (mg)	5500	800	1100
Amino acids content as is			
Alanine, g	5.76	5.18	3.72
Arginine, g	6.19	1.91	6.41
Asparagine, g	11.6	10.7	10.6
Cycteine (Cystin), g	0.76	2.16	1.01
Glutamine, g	14.8	17.5	16.7
Glycine, g	4.47	1.37	3.64
Histidine, g *	2.42	1.47	2.30
Isoleucine, g *#	5.04	6.14	3.96
Leucine, g *#	8.17	10.3	6.88
Lysine, g *	9.33	9.24	5.46
Methionine, g *	2.90	2.33	1.18
Phenylalanine, g *	4.61	2.74	4.57
Proline, g	3.62	6.19	4.63
Serine, g	4.09	4.58	4.70
Threonine, g *	4.97	7.23	3.49
Tyrosine, g *	4.33	2.89	3.64
Valine, g *#	5.37	5.60	4.16
Tryptophan	1.19	1.67	1.26
TAA	99.62	99.20	88.31
EAA*	47.14	47.94	35.64
NEAA	18.58	22.04	15.00
BCAA#	52.48	51.26	52.67

¤ Protein content evaluated by Kjeldahl methods (N*6.25), (N*6.38 for whey protein); TAA, total amino acids (true protein: Sum of all amino acids); EAA, total essential amino acids; NEAA, total non-essential amino acids; BCAA, total branched-chain amino acids. * Essentiel amino acids; # branched-chain amino acids
